# Gingival crevicular fluid levels of prolactin in periodontitis patients with controlled versus uncontrolled type 2 diabetes. a controlled clinical trial

**DOI:** 10.1038/s41598-026-68268-1

**Published:** 2026-09-07

**Authors:** Sara I. Eid, Zahraa M. Ahmed, Olfat G. Shaker, Naglaa El-Wakeel

**Affiliations:** 1 Ministry of Health, Department of Oral Medicine, Periodontology, Oral Diagnosis and Radiology, Faculty of Dental Medicine for Girls, Al-Azhar University, Cairo, Egypt; 2 Oral Medicine, Periodontology, Oral Diagnosis and Radiology Department, Faculty of Dental Medicine for Girls, Al-Azhar University, Cairo, Egypt; 3 Medical Biochemistry and Molecular Biology, Faculty of Medicine, Cairo University, Cairo, Egypt

**Keywords:** Gingival crevicular fluid, Prolactin, Type 2 diabetes mellitus, Periodontitis, Non-surgical periodontal treatment, Inflammation, Biomarkers, Diseases, Endocrinology, Medical research

## Abstract

This study investigated gingival crevicular fluid (GCF) levels of prolactin hormone (PRL) in stage III periodontitis (**P**) patients with type 2 diabetes mellitus (T2DM) before and 3 months after non-surgical periodontal treatment (NSPT). In this case-controlled trial, 80 subjects were divided into 4 groups: group I: healthy control; Group II: systemically healthy stage III **P** patients; Group III: stage III **P** patients with controlled T2DM; Group IV: stage III **P** patients with uncontrolled T2DM. All **P** patients received NSPT. Three months after NSPT, PRL levels were assessed using enzyme-linked immunosorbent assay (ELISA) as a primary outcome. Secondary outcomes were: pocket depth, clinical attachment loss, and gingival index measurements. At baseline, group IV showed the highest mean PRL levels, followed by Groups III and II, whereas group I showed the least mean values (*p* < 0.001). After 3 months, NSPT significantly improved all tested periodontal parameters except the gingival index (*p* < 0.001). NSPT reduced the PRL GCF levels in all **P** groups, with groups II and III showing significantly lower mean PRL levels than group IV (*p* < 0.001). PRL levels in groups II and III were significantly higher than those observed in group I at baseline. Elevated GCF levels of PRL seem to be linked to the disease process of **P** associated with T2DM. NSPT significantly reduced these levels. PRL could represent a valuable biomarker for periodontal inflammation associated with T2DM.

**Trial registration: **Clinicaltrials.gov. Identifier NCT06941246.on 23 April 2025.

**Supplementary Information:**

The online version contains supplementary material available at https://doi.org/10.1038/s41598-026-68268-1.

## Introduction

Prolactin (PRL) is a 199-amino-acid-long polypeptide hormone with diverse biological functions. PRL acts systemically as a hormone, with a recognized role in lactation, and locally as a cytokine with both autocrine, endocrine, and paracrine effects^1,2^. A number of biological effects are attributed to PRL, including metabolism, inflammation, and immune-modulation, causing PRL to be involved in the pathogenic mechanisms of many immune-mediated inflammatory diseases like systemic lupus, multiple sclerosis, cancer, and recently, periodontitis^3^. PRL also operates as a part of a neuroendocrine-immune network by stimulating the release of specific cytokines.

PRL and prolactin receptor (PRLR) expressions were displayed in several cell populations, including keratinocytes, fibroblasts and a wide variety of immune-related cells^4^. Previous data have documented that PRL affects metabolic homeostasis by regulating key enzymes and transporters related to insulin resistance. High PRL levels in serum indicate a high risk of obesity and dysmetabolism, such as decreased insulin sensitivity, abnormal glucose tolerance or progressive insulin resistance^5^. Taken together, these findings indicate that PRL may serve as a biological link between immune-inflammatory and metabolic dysregulation, with potential relevance to both DM and periodontal disease pathogenesis^3^.

Periodontitis (**P**) is a chronic destructive inflammatory disease of tooth-supporting structures caused by gram-negative bacteria in a microbial biofilm dysbiosis, with multi-factorial contribution. Bacterial- derived factors stimulate a local inflammatory reaction, cytokine release, followed by recruitment and activation of T and B lymphocytes causing periodontal tissue destruction and eventual tooth loss. Treatment of **P** aims mainly to reduce the microbial load and associated inflammation by mechanical debridement. However, due to the multifactorial nature of **P**, standard treatment may not be sufficient to achieve long-term clinical improvements^6–8^.

Previous data have shown that hormones can impact the pathogenic mechanisms of periodontitis. Puberty, menstrual cycle, menopause and pregnancy are all biological phases that affect periodontal health in females^9^. Further, the link between some systemic diseases associated with hormonal disturbance and periodontitis has been listed in the latest classification of Periodontal Diseases and Conditions^10^.

Diabetes mellitus (DM) is a chronic disorder caused by either inadequate synthesis of insulin by the pancreas or ineffective utilization of insulin by body tissues. Uncontrolled DM causes hyperglycemia that over time seriously damages numerous tissues and organs. Type 1 and 2 DM are the main subtypes, each with different pathophysiology, presentation, and management. Owing to the rapid increase in prevalence, DM represents an international health epidemic. Observational data suggest that **P** is associated with type 2 diabetes (T2DM) and contributes to diabetes-related complications. This association was highlighted in a recent WHO statement^11–13^.

DM is a major risk factor for periodontitis however; the underlying molecular mechanisms remain unclear. DM promotes the inflammatory/host immune response of the periodontal tissues and the pathogenicity of the periodontal microorganisms that thrive in the enriched glucose environment, altering the bacteria composition within the biofilm thereby enhancing the development of periodontitis^5^. Further, the hyperglycemia enhances oxidative stress, which exacerbates periodontal tissue destruction. Furthermore, the epigenetic changes in periodontal tissue induced by diabetes may also contribute to periodontitis^14^.

We previously hypothesized that PRL hormone might be a link involved in the association between **P** and rheumatoid arthritis^15^, and supporting evidence was provided by the observed increase in its local levels in GCF and synovial fluid of **P** patients suffering from rheumatoid arthritis (RA) compared to controls^16^.In this study, we hypothesized that PRL plays a role in the bidirectional relationship between **P** and T2DM, through its elevated local levels in GCF of **P** patients with T2DM compared to controls. Thus, this work aims to investigate local PRL levels in the GCF of patients with stage III periodontitis with controlled and uncontrolled T2DM as compared to controls. Furthermore, the effect of NSPT on these levels was also evaluated.

## Materials and methods

### Study design, outcomes and ethical approval

In this case-controlled trial, subjects were recruited from the outpatient clinic of the Oral Medicine and Periodontology Department, Faculty of Dental Medicine for girls, Al-Azhar University, from October 2024 to April 2025. Study outcomes were GCF levels of PRL hormone in controlled versus uncontrolled type 2 DM patients with stage III periodontitis, all compared to controls (primary outcome), secondary outcomes were Clinical periodontal parameters, including gingival index, probing depth, and clinical attachment loss, assessed before and after treatment. The study was conducted according to the Declaration of Helsinki (1964, revision 2008). All subjects participated voluntarily and received all information about the study. A written informed consent was obtained from each participant. This trial- due to lack of awareness- was registered retrospectively at www.ClinicalTrials.gov (NCT06941246) on 23 April 2025, and the study protocol was approved by the Ethics Committee of Faculty of Dentistry for girls, Al-Azhar University (REC-ME-25-07). A detailed medical history of each participant was obtained according to the modified Cornell Medical Index questionnaire^17^, then patients had full dental and medical examinations.

### Sample size calculation

According to previous studies^16,18^, with a primary outcome of crevicular fluid levels of PRL in periodontitis patients, a minimum total sample size of 80 participants were considered to be sufficient to detect the effect size of 0.22, with a power (1-β = 0.90) to compensate for possible dropouts and at a significance probability level of *p* ≤ 0.05. Sample size was determined using Bivariate Normal Distribution Model (G power statistical analysis program, Version 3.1.9.4).

### Patients groups

Eighty Participants were assigned equally into 4 groups Group I: healthy controls; Group II: systemically healthy stage III **P** patients; Group III: stage III **P** patients with controlled T2DM, and group IV: stage III **P** with uncontrolled T2DM.

### Inclusion and Exclusion criteria

Included subject’s age ranged from 18 to 60 years old, both genders, for group 1 (Healthy control): Systemically and periodontally healthy individuals with no bleeding on probing (BOP = 0), no clinical attachment loss (CAL = 0), and probing pocket depth (PPD) < 3 mm. For p groups, patients diagnosed with Stage III periodontitis confirmed by having interdental clinical attachment loss (CAL) ≥ 5 mm, probing depth (PD) ≥ 6 mm, radiographic bone loss extending to the middle third of the root and beyond^10^. Motivated patients with good oral hygiene.

Exclusion criteria included: pregnancy or lactation; any other known systemic disease; any previous periodontal treatment in last 6 months; antibiotics or NSAIDs in the past 3 months; smoking.

One internal medicine doctor (S.E.) did the medical examination, one periodontist (Z.S.) did the periodontal examination and GCF sampling and one periodontist (S.E.) who did the NSPT for all patients, all three examiners were blinded from each other for the diabetic, periodontal conditions of subjects and for study groups. Calibration exercises for probing measurements were performed in five patients before the study, with kappa value of 0.81 for PD and 0.78 for CAL. Laboratory assessments were conducted by O.S. who was blinded to the study groups and the statistician was blinded to group allocation when performing the analyses.

### Medical examinations

At baseline, diabetes duration and BMI for diabetic patients were recorded. Diabetic subjects provided a blood sample to determine glycemic control, assessed by levels of glycated hemoglobin (HbA1c). Non-diabetics have HbA1c levels around 5.5% T2DM patients with HbA1c levels of < 7.0% show good glycemic control and levels above > 7 indicate poor glycemic control^19^.

### Periodontal examination and treatment

Clinical and radiographic examinations were performed for all study subjects and periodontal measurements were recorded to all teeth excluding the third molars and implants; clinical attachment loss (CAL), probing pocket depth (PD), and gingival index^20^.

Measurements were recorded at six sites per teeth (mesio-buccal, mid-buccal, disto-buccal, mesio-lingual, mid-lingual, and disto-lingual) using a UNC-15 periodontal probe (Hu-Friedy, Chicago, IL, USA).

After initial examination and GCF sampling, oral hygiene instructions and prophylaxis sessions were performed with subsequent evaluation of patients’ compliance. Full-mouth NSPT was performed over two sessions using sonic, ultrasonic, and hand instruments (Gracey curettes, Hu-Friedy, Chicago, IL, USA). Treated pockets were then thoroughly rinsed with 0.2% chlorhexidine digluconate solution. Patients were instructed to rinse twice daily for 2 min with a 0.2% chlorhexidine digluconate solution for 1 week to enhance plaque control and reduce microbial recolonization during the early healing phase following NSPT^21^.

### Gingival crevicular fluid sampling

At baseline, GCF samples were collected from all subjects and 3 months after NSPT (except for group I). GCF samples were obtained from the buccal aspect of interproximal site related to teeth with the greatest signs of inflammation and attachment loss, by placing filter paper strip (Periopaper; ProFlow Inc., Amityville, NY, USA) in the selected sites for 30 s. The selected site was isolated with cotton rolls and samples were collected after cautious removal of supragingival deposits. For the periodontally healthy controls, samples were collected from the upper first molar and blood contaminated strips were discarded. After collection, samples were immediately centrifuged and stored at − 80 °C until further analysis. Samples were assayed for PRL by using ELISA kits (Prolactin levels in gingival crevicular fluid were measured using a commercial enzyme-linked immunosorbent assay (ELISA) kit (Pishtaz Teb Diagnostics, Germany; according to the manufacturer’s instructions. The assay is based on a sandwich ELISA principle in which prolactin is captured between a solid-phase monoclonal antibody and an HRP-conjugated detection antibody. Absorbance was measured at 450 nm, and concentrations were calculated using a standard curve (Prechek Bio, Inc. CA, USA). Results were presented as concentrations in nanograms per milliliter (ng/mL); sensitivity of the assay was 5 ng/mL.

### Statistical analysis

Numerical data were explored for normality by Kolmogorov–Smirnov and Shapiro–Wilk tests. All data showed parametric distribution except for gingival index. Data were presented as mean and standard deviation (mean ± SD) values. For parametric data, one-way ANOVA and repeated measures ANOVA tests were used to compare between the groups as well as to study the changes after treatment within each group. Bonferroni’s post hoc test was used for pair-wise comparisons when ANOVA test was significant. For non-parametric data, Mann–Whitney *U* test was used to compare between periodontitis groups. Wilcoxon signed-rank test was used to study the changes within each group. Qualitative data were presented as frequencies and percentages. Chi-square test was used to compare between the groups. The significance level was set at *P* ≤ 0.05. Statistical analysis was performed with IBM SPSS Statistics for Windows, version 23.0. Armonk, NY: IBM Corp.

## Results

### Baseline characteristics

Eighty participants aged 18–59 years were included. Males and females were equally represented in the study. In Groups III and IV, mean ± SD for duration of diabetes was 44 ± 21.2 and 27.5 ± 10.8 months, respectively. The results showed significant differences between groups in terms of age, HbA1c, and BMI. The mean age was highest in Group IV (48.35 ± 7.48 years) followed by Group III (46.20 ± 6.44 years), then Group II (45.30 ± 9.39 years), and lowest in Group I (29.50 ± 4.81 years). For HbA1c, Group IV had a higher mean value (9.64 ± 0.83) compared to Group III (6.68 ± 0.18). Regarding BMI, Group IV had the highest mean value (28.97 ± 2.03) followed by Group II (28.32 ± 2.18), then Group III (27.89 ± 2.50), and lowest in Group I (24.60 ± 1.72). These differences were statistically significant (*p* < 0.001) (Table 1).

**Table 1:** Comparison between groups according to demographic data..

Demographic data	GI (*n* = 20)	GII P (*n* = 20)	GIIIC (*n* = 20)	GIVUC (*n* = 20)	Test value	*p*-value
**Age “years”**						
Mean ± SD	29.50 ± 4.81^**B**^	45.30 ± 9.39^**A**^	46.20 ± 6.44^**A**^	48.35 ± 7.48^**A**^	28.692	0.001*
Range	18–35	26–58	37–59	35–59		
**HA1C**						
Mean ± SD	--	--	6.68 ± 0.18	9.64 ± 0.83	245.116	0.001*
Range	--	--	6.2–6.9	8–10.8		
**BMI [wt/(ht)^2]**						
Mean ± SD	24.60 ± 1.72B	28.32 ± 2.18 A	27.89 ± 2.50 A	28.97 ± 2.03 A	16.841	0.001*
Range	21.6–28.2	25.49–34.1	23.5–31.2 A	26.2–33 A		
**Gender Male** **Female**	(50%)10 10 (50%) A	(50%)10 10 (50%) A	(50%)10 10(50%) A	10(50%) 10(50%) A	0,000	1.000

GI: group1 Healthy (Control); GII** P**: group 2Stage III periodontitis without diabetes; **GIIIC**: group3 Stage III periodontitis with controlled type 2 diabetes.

**GIVUC**: group4 Stage III periodontitis with uncontrolled type 2 diabetes; **(n=20 **number of participants.

Different capital letters indicate significant difference at (*p*<0.05) among means in the same row.

P-value >0.05 is insignificant; *p-value <0.05 is significant.

BMI: Body Mass Index., HbA1C; Hemoglobin A 1C, P value (effect of time).

### Prolactin levels before and after non‑surgical periodontal therapy

At baseline, there was a statistically significant difference between all tested groups. Group IV showed the highest mean PRL values (56.71 ± 9.00), followed by Group III, then Group II, whereas Group I showed the lowest mean PRL levels (11.25 ± 0.78) (*P* < 0.001, effect size = 0.911). After treatment, all **P** groups (Groups II, III, and IV) showed a statistically significant reduction compared to pretreatment levels within each group (*P* < 0.001). Between-group comparisons showed no significant difference between Groups II and III in the post-treatment PRL levels (21.99 ± 1.88 and 20.74 ± 3.36 respectively; *P* > 0.05). Both groups showed significantly lower post-treatment PRL levels than Group IV (*P* < 0.001) (Table 2).

**Table 2:** Comparison between groups according to all variables.

	GI	GII	GIII	GIV	*p*-value
**Pocket Depth (PD) **					
T0	1.50±0.43B	5.87±1.35 A	6.20±0.80 A	6.10±1.31 A	0.001*
T3	----	4.55±1.19B	5.15±0.89 A	5.45±1.13 A	0.001*
P value (effect of time)	----	0.001*	0.001*	0.001*	----
**Clinical attachment Loss (CAL)**					
T0	0.63±0.39B	5.43±0.41 A	5.35±0.54 A	5.55±0.65 A	0.001*
T3	----	4.23±0.57B	4.30±0.68B	4.90±0.62 A	0.001*
P value (effect of time)	----	0.001*	0.001*	0.001*	----
**Gingival Index (GI)**					
T0	0.15±0.07 B	2.60±0.50 A	2.45±0.60 A	2.70±0.47 A	0.001*
T3	----	1.40±0.40 B	1.65±0.45 B	2.50±0.51 A	0.001*
P value (effect of time)	----	0.001*	0.001*	0.104	----
**Prolactin hormone**					
T0	11.25±0.78 D	46.75±6.72 C	51.38±5.91 B	56.71±9.00 A	0.001*
T3	----	21.99±1.88 B	20.74±3.36 B	23.95±3.17 A	0.001*
P value (effect of time)	----	0.001*	0.001*	0.001*	----

GI: group 1Healthy (Control); GII: group 2 Stage III periodontitis without diabetes; GIII: group 3Stage III periodontitis(P) with controlled type 2 diabetes (T2DM).

GIV: group 4 Stage III periodontitis with uncontrolled type 2 diabetes; T0: Examination at baseline, T3: Examination after 3 months postoperatively.

Different capital letters indicate significant difference at (*p*<0.05) among means in the same row.

*P*-value >0.05 is insignificant; *p-value <0.05 is significant PD: Pocket Depth, CAL: Clinical attachment Loss, GI: Gingival Index, PRL: Prolactin hormone, P value (effect of time).

### Clinical parameters

At baseline, no significant differences were observed among the 3 periodontitis groups in all tested parameters (PD, CAL, and GI) and all values were statistically higher than those of the control group. ***After treatment***, the tested periodontal parameters showed a significant reduction in all **P** groups (Groups II, III, and IV) (*P* < 0.001) except for the gingival index in Group IV that showed a non-significant change after treatment (*P* = 0.104). **For PD**, Group IV (5.45 ± 1.13 mm) had the highest mean PD value followed by group III (5.15 ± 0.89 mm), then group II (4.55 ± 1.19 mm), and the lowest value was in group I (1.50 ± 0.43 mm)((*p* < 0.001).For PD intra-group comparison, groups II, III, and IV after 3 months, with mean differences of −1.33 ± 0.30 (*p* < 0.001), −1.05 ± 0.24 (*p* < 0.001), and − 0.65 ± 0.15 (*p* < 0.001), respectively. For, CAL, a significant difference between groups was reported, Group IV (4.90 ± 0.62 mm) had the highest mean value followed by group III (4.30 ± 0.68 mm) and group II (4.23 ± 0.57 mm), and the lowest value was in group I (0.63 ± 0.39 mm) (*p* < 0.001). For CAL intra-group comparison, significant reductions were seen in groups II, III, and IV after 3 months, with mean differences of −1.20 ± 0.28 (*p* < 0.001), −1.05 ± 0.24 (*p* < 0.001), and − 0.65 ± 0.15 (*p* < 0.001), respectively (Table 2).

## Discussion

**Fig. 1:**
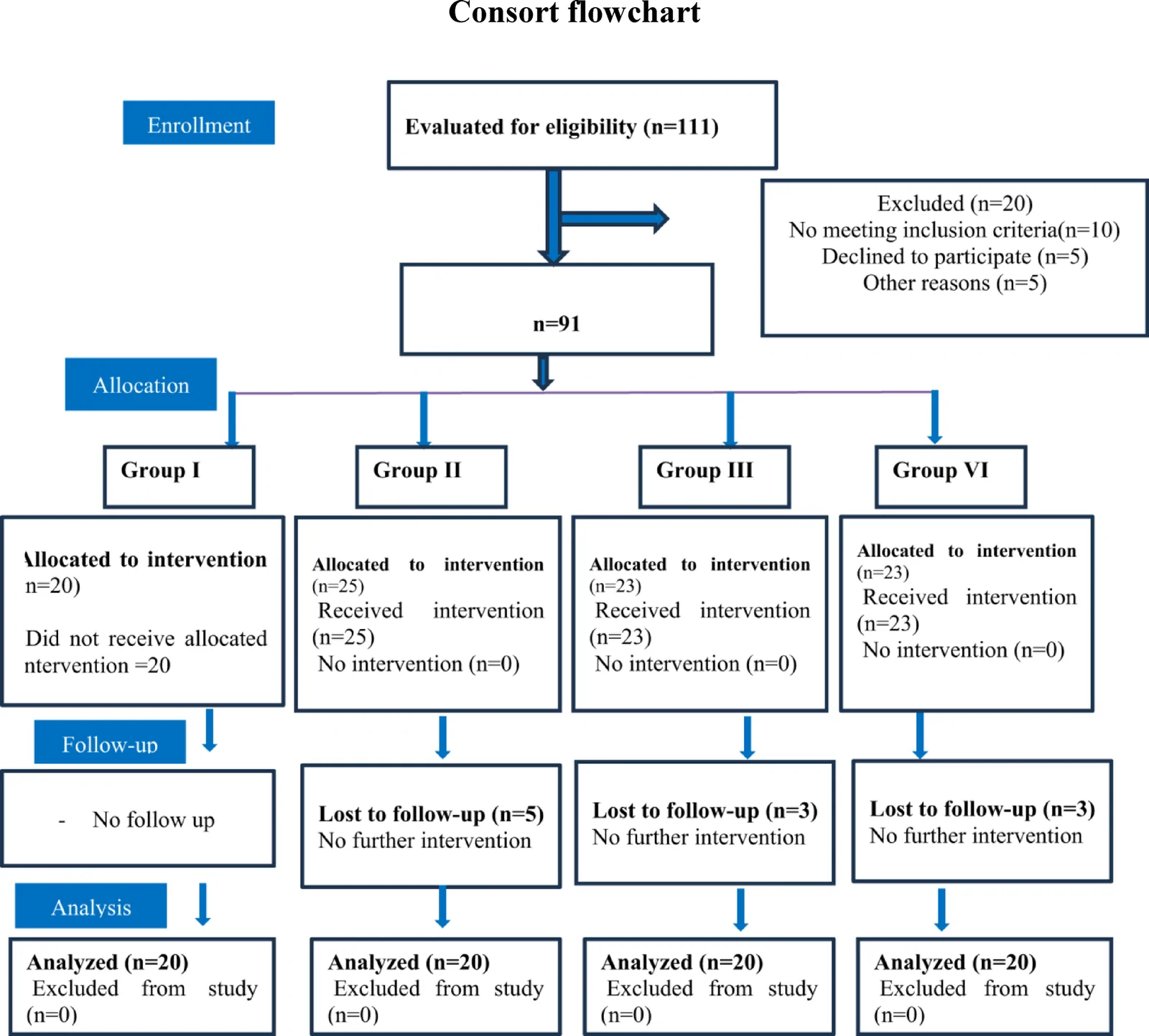
Flowchart for subjects allocation to groups and treatment.

The known two-way relationship between DM and periodontitis is attributed to the elevated systemic inflammatory burden, with diabetes increasing the risk for periodontitis, and periodontal inflammation negatively affecting glycemic control. However, the exact nature of this association is not fully understood^22^. This case-controlled study reports, for the first time, local gingival crevicular fluid levels of PRL hormone in well-defined groups of patients with periodontitis (**P**)and controlled or uncontrolled T2DM. Furthermore, the effect of non-surgical periodontal therapy on these levels was evaluated (Fig. 1).

At baseline, the uncontrolled T2DM + **P** patients had non-significantly worse periodontal clinical parameters as compared with the other periodontitis groups, in accordance with^23,24^. Previous data had shown significantly worse periodontal indices (community periodontal index and CAL) in uncontrolled T2DM patients compared to controlled patients and healthy controls. However, this study reported a non-significant difference in gingival index that was almost similar among the three studied **P** groups, this is consistent with previous data. In further agreement with our baseline findings, recent work reported the increased prevalence of the CAL (range 4–5 mm) in the non-diabetic **P** patients, followed by controlled T2DM and the uncontrolled T2DM had the least prevalence of the 4–5 mm CAL. However, this study reported the increased prevalence of the above 8 mm CAL among the uncontrolled T2DM, followed by the controlled T2DM followed by the non-diabetic periodontitis patients^25^. The previously reported similarities and discrepancies in the tested periodontal indices in **P** patients with T2DM could be attributed to factors such as sociodemographic characteristics, study design, severity of diabetic condition, medications taken, sample size, oral hygiene behavior, the studied periodontitis parameter, education level, and access to dental services.

In this work, diabetic patients treated with NSAIDs were excluded to avoid their possible effect on the inflammatory status and PRL expression as previously mentioned^26–28^. To further clarify the link between glycemic status and inflammation-associated PRL levels before and after NSPT, patients with T2DM were separated into 2 groups with good and poor glycemic control according to the HbA1c levels.

For post-treatment outcomes, all intervention groups showed a significant improvement in all tested periodontal parameters (except for GI in group IV) after NSPT, in accordance with known effects of NSPT in alleviating periodontal inflammation in T2DM patients^29–32^. After treatment, CAL and GI values were non-significantly different between the healthy **P** and the controlled T2DM groups, both were significantly better than the uncontrolled T2DM group. The non-significant improvement of GI after NSPT in the fourth group could be attributed to reduced adherence to plaque-control measures as well as poor glycemic control As for the PD values, the two T2DM groups were non-significantly worse compared to the periodontitis group, agreeing with earlier reported data^30,32,33^. The non-significant difference reported for PD values after NSPT in both diabetic groups with a significantly lower PD reduction compared to **P** group was in agreement with earlier study^32^. However, CAL is considered the gold standard in predicting the severity of periodontal destruction^34^.

The possible association between prolactin and T2DM was long reported and recently re-investigated, but only on its systemic levels, however, evidence for a casual relation between T2DM, glycemic control and systemic PRL levels are still extremely lacking^35–38^. Recent data reported that increased PRL levels within the normal range was associated with a reduced risk of T2DM^36,39^. Apart from the systemic effects of PRL that might be influenced by various factors, the identification of PRL receptors in various tissues has shifted our interest, along with others, to the effects of locally produced PRL in diseased tissues activating PRLR on target cells^16,40,41^.

In agreement with our results, the association between elevated locally produced PRL in GCF with periodontitis-associated inflammation and the significant reduction of those levels after periodontal treatment was earlier reported^3,16^. Further, recent work showed that salivary PRL levels were positively associated with P and these levels were reduced significantly after NSPT adding more support to the cytokine effects of local PRL release in periodontal inflammation^42^. In this work, PRL levels were highest in GCF of uncontrolled T2DM + P patients compared to controlled T2DM periodontitis patients and both were greater compared to P patients without DM and healthy controls, providing further evidence of the association of PRL with periodontal inflammation associated with systemic diseases.

Insulin resistance associated with T2DM impacts immune cells (T and B cells, macrophages, and neutrophils), causing elevated levels of pro-inflammatory cytokines (TNF-α, IL-6) and adipokines (leptin, resistin), this, in turn, exacerbate insulin resistance and promote a vicious cycle of metabolic and immune dysregulation. This interplay contributes to the chronic low-grade inflammation associated with T2DM pathogenesis, and further impairs insulin signaling and glucose metabolism^43^. The elevated GCF levels of PRL in the uncontrolled diabetic group in this work could be attributed to the combined inflammatory effect of periodontal disease and T2DM-associated insulin resistance on local immune responses.

Post-treatment PRL levels were higher in all periodontitis groups than those observed in healthy controls, indicating the chronic nature of periodontitis^44^. The elevated crevicular expression of PRL in periodontitis can be attributed to mediators of periodontal tissue destruction, such as IL-1 and TNF-alpha, that are well known stimulators of PRL secretion; however, this needs to be supported by further in vivo and in vitro studies to prove this explanation^45^. Our work further supports the clinical applicability of evaluating PRL in GCF sampling as a non-invasive, simple, and readily available method for monitoring disease activity and response to treatment.

We earlier proposed that PRL might play a role in the mechanistic interaction between P and RA^15^, and this was partially supported by our later findings^16^. In this work, the role of prolactin in periodontal inflammation associated with systemic inflammatory diseases is reinforced, suggesting that PRL might be involved in the mechanisms underlying the association between T2DM and **P**, but more data are needed to explore the possible intervening mechanisms. In this regard, we can propose a hypothesis for a possible mechanism underlying this association that can be put to be investigated, for example, it has recently been reported that PRL activates JAK2/STAT, MAPK, and PI3K/AKT signaling pathways, boosting immune cell proliferation, survival, and angiogenesis^46^. Interestingly, insulin signaling plays a crucial role in immune cell activation, proliferation, and inflammation regulation, primarily through the PI3K/Akt and MAPK pathways. For instance, in macrophages, insulin binding activates the IRS/PI3K/Akt pathway, which is essential for glucose uptake and inflammatory response modulation, but insulin resistance associated with T2DM disrupts this pathway, leading to chronic inflammation^43^. Another possible mechanism may involve the enhanced oxidative stress associated with periodontitis, T2DM, and local PRL secretion^47–49^. The above-mentioned findings can be of value when we try to explain why local PRL levels are associated with DM and periodontitis, however, this requires further investigation.

### Study strengths and limitations

A major strength of this study is its case-controlled design, that included well-characterized groups of subjects (stage III periodontitis patients with controlled and uncontrolled T2DM all compared with systemically and periodontally healthy subjects). However, limitations include the relatively small sample size, retrospective registration at ClinicalTrials.gov, and the non-randomized design that is more susceptible to bias. Further, some obese subjects and a wide age range of participants were included in group IV in this work, which might affect the interpretation of results. Thus, in light of the limitation of this work, more randomized controlled clinical trials with larger sample sizes and longer follow-up periods are needed to validate our results.

## Conclusions

This work highlights the potential role of prolactin as a cytokine and biomarker for periodontal inflammation associated with T2DM. Moreover, non-surgical periodontal treatment improved the tested clinical parameters and reduced PRL levels, thus, PRL also can predict (serve as a potential marker) response to treatment. This work is the first to report the effect of glycemic control on local periodontal PRL levels. However, the mechanisms underlying the increased expression of PRL in inflamed periodontal tissues, and in different stages of disease development remain poorly understood. Also, the possible confounders that affect the activity of PRL such as cell type, PRL concentration, PRLR isoform and cytokines in the medium, as well as systemic factors such age, obesity, other hormones, all need further investigation.

## Supplementary Information

Below is the link to the electronic supplementary material.

Supplementary Material 1

Supplementary Material 2

Supplementary Material 3

## Data Availability

Data available on request Duo to privacy/ethical restrictions.
