# Supplementary material for: Gingival crevicular fluid levels of prolactin in periodontitis patients with controlled versus uncontrolled type 2 diabetes. a controlled clinical trial
Source: Sci Rep. 2026 Sep 7;16:27945. doi: 10.1038/s41598-026-68268-1 (PMC13550468; doi:10.1038/s41598-026-68268-1)
Supplement: Supplementary file 2 [file 41598_2026_68268_MOESM2_ESM.pdf]

# Gingival Crevicular Fluid Levels of Prolactin Hormone in Type 2 Diabetic Patients with Stage III Periodontitis Before and After Non-Surgical Treatments

**A proposal**

Submitted to the Faculty of Dental Medicine for Girls, Al- Azhar University in Partial Fulfillment of the Requirement for Master Degree

**In**

**Oral Medicine, Periodontology, Oral Diagnosis and Radiology**

**By**


**Sara Ibrahim Hussein Eid**

B.D.S (2017) Al-Azhar University

Dentist at Aga central hospital



Faculty of Dental Medicine for Girls

Al-Azhar University

2024 G - 1445 H

**OMPDR_105_1p**

## Supervisors


**DR. Naglaa Mohamed El-Wakeel**

Professor and head of Oral Medicine, Periodontology, Oral Diagnosis and Radiology Department

Faculty of Dental Medicine for Girls

Al-Azhar University

**DR. Olfat Gamil Shaker**

Professor of Medical Biochemistry and Molecular Biology

Faculty of Medicine

Cairo University

**DR. Zahraa Mohamed Ahmed Saeed**

Lecturer of Oral Medicine, Periodontology, Oral Diagnosis and Radiology Department

Faculty of Dental Medicine for Girls

Al- Azhar University

# Introduction

Periodontitis is a chronic inflammatory multifactorial disease affecting the tooth supporting structures and is associated with dysbiotic plaque biofilms resulting in progressive clinical attachment loss and alveolar bone destruction, three forms of periodontitis can be identified: necrotizing periodontitis, periodontitis as a manifestation of systemic disease and periodontitis. Staging involves four categories (stage1 through 4). Grading include three levels (grade A- low risk, grade B- moderate risk, grade C – high risk for progression) (1).

The destructive procedures of periodontitis develop out of interplay between the dysbiotic community and imbalanced host immune response, in addition to genetic, systemic, and environmental modifying risk factors leading to destruction of periodontal tissues, in addition to acting as a tempering factor of the systemic health of the patient (2).

Diabetes mellitus, a prevalent chronic metabolic disorder, encompasses types like Type 1 (T1DM), Type 2 (T2DM), and gestational diabetes. Type 2 diabetes mellitus (T2DM) is a metabolic disorder characterized by chronic hyperglycemia with multiple etiologies, yet its full etiology remains incompletely understood. The primary manifestations include poor insulin secretion, low insulin sensitivity, and insulin resistance (IR) (3).

Given the high morbidity and mortality associated with diabetes mellitus (DM) and its various complications such as cardiovascular disease (CVD), diabetic nephropathy (DN), and oral diseases, DM has emerged as a global health concern (4). The most significant challenge for medical practitioners currently is the effective control of blood sugar. Prolonged

hyperglycemia can lead to periodontal tissue lesions in patients, with scholars identifying periodontitis (PD) as the sixth major complication of DM in 1993 [5].

Periodontitis is associated with an array of systemic diseases and conditions such as diabetes mellitus (DM). Both are chronic diseases, with common etiopathogenic and pathophysiological associations. The relationship between periodontal disease and DM is bidirectional. Periodontal disease is currently considered the sixth complication of DM, and periodontitis affects the prevalence, progression, and therapeutic management of DM [6].

It has been demonstrated that hormones can affect periodontal pathogenic mechanisms. Puberty, menstrual cycle, menopause and pregnancy are all biological phases that affect periodontal health in females [7]. Further, the link between some systemic diseases associated with hormonal disturbance and periodontitis has been defined in the latest classification of Periodontal Diseases and Conditions [8].

Prolactin (PRL) is a 23-kD peptide hormone secreted in the pituitary gland, in extra-pituitary locations, such as adipose tissue, and produced by immune cells [9]. Although PRL's main function is to regulate differentiation and lactation of the mammary epithelium, it also plays an important regulatory role in inflammation, cell survival, and proliferation by binding to the PRL receptor (PRLR) - a member of the type 1 cytokine /hematopoietic receptor superfamily- which is widely expressed through the immune system cells, including monocytes, lymphocytes, macrophages, natural killer cells and granulocytes [10,11].

The increased local production of PRL at sites of active inflammation and subsequent tissue destruction has been reported. For

example, PRL levels are increased in the synovial fluid levels in patients with active RA compared to controls, suggesting that these elevated levels are due to increased local secretion by immune cells in relation to disease activity [12].

Gingival Crevicular Fluid (GCF) offers a non-invasive, efficient and easy to use approach to sample biomarkers of inflammation and bone resorption in the oral cavity and allow to differentiate the active inflamed sites and predict future tissue destruction and to diagnose early signs of periodontitis. Furthermore, it can also allow monitoring of this condition and prompt discovery of new biomarkers that will aid in the development of new therapeutic approaches via host modulatory drugs for periodontal disease treatment leading to more individualized, targeted treatments for oral health [13].

Periodontitis and DM are common diseases in modern societies that have negative effects on health and quality of life. As evident in recent research, both are highly prevalent and expected to increase. Thus, seeking newer biomarkers that could help to establish the most appropriate prevention and treatment modalities is the challenge of research nowadays [6].

Oral hygiene instructions only resulted in a better gingival condition of diabetic patients in the initial time. The nonsurgical periodontal therapy by combining scaling and root planning (SRP) and oral hygiene instructions (OHI) significantly improved both periodontal health and glycemic control [14]. Non-surgical periodontal therapy is a critical aspect of periodontal disease management, targeted at the removal of the etiologic factor, thereby arresting disease progression and the re-establishment of biologically acceptable root surfaces for healing [15].

Periodontal treatment in diabetic patients reduces systemic inflammatory burden and hence should be closely coordinated with the patient's overall clinical diabetic management. In a study involving 371 T2DM patients receiving basic periodontal treatment, HbA1c levels decreased by an average of 0.40 % at 3–9 months of follow-up, suggesting that effective periodontal treatment contributes to blood glucose control to a certain extent [16].

To the best of our knowledge, no data elucidate the relationship between periodontitis, DM, and PRL; therefore, we intended to estimate its level to obtain an insight into the expected role of PRL in the pathogenesis of periodontitis patients with and without DM. We hypothesized that an up-regulation of local PRL levels would be present in periodontitis patients. Periodontal inflammatory factors induce excessive autophagy in pancreatic β cells, thereby reducing insulin secretion and promoting the progression of T2DM, remains unclear. To address this gap, we will conduct the present study aimed to address whether there is an increase in the local prolactin levels in diabetic patients with periodontitis compared to controls.

# Aim of the work

**The present study will be performed to:**

Investigate a potential role of prolactin hormone in the pathogenesis of periodontitis by assessing its local gingival levels in patients with type two diabetes with stage III periodontitis before and after non-surgical treatments compared to controls.

**Research objectives:**

To evaluate the role of prolactin hormone in the pathogenesis of periodontitis by assessing its local gingival levels and assess clinical parameters in patients who have type two diabetes.

**Research question:**

The question of this study is presented in the form of a PICOT question:

P (Population): patients, with type 2 diabetes, with stage III periodontitis.

I (Intervention): Non-surgical periodontal therapy.

C (Control):

- Healthy participant.

O (Outcome):

Primary outcome:

Gingival crevicular fluid levels of prolactin hormone in different groups before and after nonsurgical treatments.

Secondary Outcome:

Clinical parameters: Gingival index, plaque index, probing depth and clinical attachment levels before and after treatments.

T (Time):

A total 3 months from base line.

# Material and Methods

## I. Materials/ Armamentarium:

The present study will include the following groups:

**Group I:** Healthy (control).

**Group II:** Stage III periodontitis without diabetes

**Group III:** Stage III periodontitis with controlled type 2 diabetes

**Group IV:** Stage III periodontitis with uncontrolled type 2 diabetes

## II. Methods:

1. **Study design:**

   Comparative, prospective, clinical trial.

2. **Study area:**

   The patients will be selected from those attending to the Out-Patient Clinic of Oral Medicine, Periodontology, Oral Diagnosis and Radiology department, Faculty of Dental Medicine for Girls, Al-Azhar University.

3. **Study subjects:**

   Patients will be selected according to the following inclusion and exclusion criteria:

**The inclusion criteria are [17]:**

1- Age range from 18 to 60 years.

2- Stage III periodontitis.

3- Type 2 diabetes.

4- Only patients signing informed written consents.

**Exclusion criteria are [17]:**

1) Pregnancy or lactation.

2) Any known systemic disease.

3) Any type of previous periodontal treatment (surgical or non-surgical) in the preceding 6 months.

4) Smoking (former or current).

4) **Sample size calculation:**

To evaluate the role of prolactin hormone in the pathogenesis of periodontitis by assessing its local gingival levels and assess clinical parameters in patients who have type two diabetes with stage III in three major groups (GII, GIII, and GIV) beside the control group (Healthy group GI) at two measurement point (Before operative, and after 3 months ), the results will be statistically analyzed using one-way analysis of variance (ANOVA) followed by a post hoc test or an equivalent non-parametric test, and the Pearson's correlation coefficient (r) test will be used for correlating between clinical (PI, GI, PD, and CAL) and Biochemical results.

According to the previous studies [17,18], a minimum total sample size of 90 samples will be sufficient to detect the effect size of 0.22, with a power ($1-\beta=0.90$) at a significance probability level of $p \leq 0.05$ to Investigate a potential role of prolactin hormone in the pathogenesis of periodontitis by assessing its local gingival levels in patients with type two diabetes with stage III periodontitis before and after non-surgical treatments compared to controls.

According to sample size calculations, there is a 90% chance of correctly rejecting the null hypothesis of no significant effect if each group represented by 20 samples. The sample size was calculated according to G*Power software version 3.1.9.7.

**Table (1):** Variables of the study

| Variables | Symbol | Denotes for |
|---|---|---|
| **Type of group** | **GI** | Healthy (Control) |
| | **GII** | Stage III periodontitis without diabetes |
| | **GIII** | Stage III periodontitis with controlled type 2 diabetes |
| | **GIV** | Stage III periodontitis with uncontrolled type 2 diabetes |
| **Time intervals to assessment (Clinical and Biochemically) (T)** | **$T_0$** | Examination at baseline |
| | **$T_3$** | Examination after 3 months postoperatively |

**Table (2):** Interaction between variables

| Main groups | Time intervals | | Total |
|---|---|---|---|
| | **$T_0$** | **$T_3$** | |
| **GI (20 Samples)** | $GIT_0$ | $GIT_3$ | **20** |
| **GII (20 Samples)** | $GIIT_0$ | $GIIT_3$ | **20** |
| **GIII (20 Samples)** | $GIIIT_0$ | $GIIIT_3$ | **20** |
| **GIV (20 Samples)** | $GIVT_0$ | $GIVT_3$ | **20** |
| **Total** | | | **80** |

## 5) **Intervention**

### ***Periodontal Examination***

At baseline, all participants received a full-mouth clinical examination, and the following periodontal parameters will be recorded: Plaque Index (PI) (19), Gingival Index (GI) (20), Pocket Depth (PD), and Clinical Attachment Loss (CAL).

Initial periodontal examination, full mouth scaling and root planning (SRP), clinical measurements before and after treatment, gingival crevicular fluid sampling as well as reinforcement of oral hygiene at

baseline and during follow-up evaluations will be performed by the same periodontist (E.A.). Reinforcement of oral hygiene will be done every month for 3 months.

***Gingival fluid sampling:***

Gingival crevicular fluid (GCF) collection will be done the second day after clinical examination to prevent the contamination of sample with blood associated with the probing of inflamed sites. Samples will be collected from the buccal aspects of two interproximal sites in teeth that had the highest signs of inflammation and attachment loss for periodontitis groups, this will be done before, and 3 months after SRP. For the control group, samples will be collected from the upper first molar.

The samples will be assayed for PRL by using an enzyme linked immunosorbent assay (ELISA) kits.

**Patient's examinations:**

Glycosylated Hemoglobin (Hemoglobin A1c) test and Body Mass Index will be measured to all participants.

6. **Study duration:**

The study duration will be within 3 months.

**7. Statistical analysis:**

All data will be collected, tabulated and statistically analyzed.

**8. Ethical approval:**

- This study was approved by the Research Ethical Committee (REC) of the faculty of Dental Medicine for girls, Al-Azhar University.

# <u>الموافقة المستنيرة للمتطوعين</u>

**عنوان البحث باللغة العربية:**

مستويات السائل اللثوي من هرمون البرولاكتين لدى مرضى السُكرى من النوع الثانى المصابين بالتهاب اللثة فى المرحلة الثالثة قبل وبعد العلاجات غير الجراحية

**الهدف من إجراء البحث:**

إجراء الدراسة الحالية لتقييم الدور المحتمل لهرمون البرولاكتين في التسبب في إلتهاب دواعم السن من خلال تقييم مستوياته المحلية في اللثة لدى المرضى الذين يعانون من مرض السكري من النوع الثاني مع التهاب اللثة فى المرحلة الثالثة قبل وبعد العلاجات غير الجراحية مقارنة بالضوابط.

**<u>الفائدة المباشرة للشخص المتطوع:</u>**

سيتم علاج أمراض اللثه إلتهاب دواعم السن التى لم يتم علاجها التى من الممكن أن تؤدى إلى فقدان الأسنان.

**<u>الفائدة العلمية والفائدة العامة المرجوة من البحث:</u>**

معرفة أي. دور محتمل للبرولاكتين في التسبب في أمراض اللثة وكمؤشر حيوي لنشاط المرض كذلك معرفة إن كان علاقة بين التهاب اللثة ومرض السكرى من النوع الثانى وهذا ممكن أن يساعد فى الحفاظ على الأسنان عن طريق العلاج المناسب.

**<u>الأعراض الجانبية ودرجة المخاطر المتوقع حدوثها وكيفية التعامل معها:</u>**

خطر التلوث وسيتم التعامل معها بقواعد مكافحة العدوى.

<u>:المعرفة الكاملة للمريض بخطوات البحث</u>

[ ] قراءة [ ] شرح شفهي [ ] أخرى

١. لقد اطلعت بعناية وفهمت الغرض من إجراء البحث وطبيعة هذه الدراسة، وأنا أفهم ما هو ضروري لإنجاز هذه الإجراءات.
٢. سوف يتم اجراء العلاج في كلية طب الأسنان بنات جامعة الازهر أو أي جهة معتمده مثل الجامعات الأخرى أو المراكز البحثية.
٣. قد أعلمني الطبيب الباحث بالبدائل العلاجية الممكنة لهذا البحث.
٤. لقد أبلغني الطبيب الباحث بجميع المخاطر المحتملة لهذا البحث وكيفية التعامل معها.

٥. أوافق على التصوير والتسجيل، وجميع أنواع الأشعة والتي يتعين القيام بها في هذا الدراسة، بشرط عدم الكشف عن هويتي.

٦. - لقد قدمت تقريرا دقيقا عن تاريخ حالتي الصحية. وأبلغت الطبيب بجميع أنواع ردود الأفعال الصحية أو الحساسية غير العادية من الأدوية والأغذية أو لدغ الحشرات أو مواد التخدير والغبار أو أي ردود أفعال حدثت لي من أي مواد أخرى، ونزيف غير طبيعي أو أي ظروف أخرى ذات صلة على صحتي

٧. أقر بأني غير مشترك في أي بحث أخر منذ بداية هذا البحث وحتى انتهائه وأنى سأعلم الطبيب الباحث لو دخلت أي بحث أخر طوال فترة هذا البحث.

٨. أتعهد بإعادة الأجهزة (الأدوات) الطبية المستخدمة في البحث في حالة التوقف أو عند انتهاء البحث.

بعد معرفة المعلومات المتاحة الخاصة بالبحث يتفضل الشخص المتطوع أو المسئول عنه بالاختيار بحرية ما بين الاشتراك من عدمه. في حال الموافقة يتفضل بملء البيانات الموضحة. من حق المتطوع الانسحاب من البحث بدون إبداء الأسباب مع مراعاة حق استرجاع الباحث لأي أجهزة أو أدوات طبية مستعملة بغرض البحث بحوزة المتطوع (تسمى من قبل الباحث)

تعهد الطبيب المسئول عن البحث بالتالي:

- الحفاظ على سرية المعلومات الخاصة بالشخص المتطوع بالمشاركة في البحث مع ذكر الطرق المستخدمة لذلك مثل استبدال الاسماء بأرقام كوديه أو اخفاء معالم الوجه عند التصوير الفوتوغرافي ان أمكن (إلخ ..)
- إذا حدث خطأ طبي غير مقصود فان الباحث يلتزم أن يقوم بإصلاحه بقدر الممكن

في حالة وجود شكاوى: يتم التواصل مع منسق لجنة الاخلاقيات في القسم كما يمكن التواصل مع اللجنة مباشرة عن طريق البريد الإلكتروني_

**ethics.committee@azhar.edu.eg**

منسق القسم: -------------------------------------------------------------

<u>بيانات المتطوع</u>

| | |
|---|---|
| اسم المتطوع: | تاريخ الميلاد: |
| اسم ولي الأمر أو المرافق (عند اللزوم): | الرقم القومي: |
| العنوان: | الهاتف: |
| اسم الباحث | التاريخ : |
| توقيع الباحث: | التاريخ: |
| توقيع المشرف على البحث (في حالة الرسائل): | التاريخ: |

هذا البحث تمت موافقة اللجنة عليه برقم

<u>من حق المتطوع الاحتفاظ بنسخه مصورة من الموافقة المستنيرة للبحث الذي تطوع فيه</u>

# References


1. **Papapanou PN, Sanz M, Buduneli N, Dietrich T, Feres M, Fine DH, et al.** Periodontitis: Consensus report of workgroup 2 of the 2017 World Workshop on the Classification of Periodontal and Peri-Implant Diseases and Conditions. J Periodontol. 2018 Jun; 89 Suppl 1:S173-S182.
2. **Hajishengallis G, Lamont RJ.** Polymicrobial communities in periodontal disease: their quasi-organismal nature and dialogue with the host**.** Periodontol 2000. 2021; 86:210–230.
3. **Hu Y., Zong G., Liu G., Wang M., Rosner B., Pan A., et al.** Smoking cessation, weight change, type 2 diabetes, and mortality. N. Engl. J. Med. 2018; 379:623–632.
4. **Nitta H., Katagiri S., Nagasawa T., Izumi Y., Ishikawa I., Izumiyama H., et al.** The number of microvascular complications is associated with an increased risk for severity of periodontitis in type 2 diabetes patients: results of a multicenter hospital-based cross-sectional study. J Diabetes Investig. 2017; 8:677–686.
5. **Saini R., Saini S., Sugandha R.** Periodontal disease: the sixth complication of diabetes. J Family Community Med. 2011; 18:31.
6. **Păunică I, Giurgiu M, Dumitriu AS, Păunică S, PanteaStoian AM, Martu M-A, Serafinceanu C.** The bidirectional relationship between periodontal disease and diabetes mellitus—a review. Diagnostics. 2023; 13:68.
7. **Khosravisamani M, Maliji G, Seyfi S, Azadmehr A, Abd Nikfarjam B, Madadi S, Jafari S.** Effect of the menstrual cycle on inflammatory cytokines in the periodontium. J Periodontal Res. 2014 Dec; 49(6):770-6.

8. **Jepsen S, Caton JG, Albandar JM, Bissada NF, Bouchard P, Cortellini P, et al.** Periodontal manifestations of systemic diseases and developmental and acquired conditions: Consensus report of workgroup 3 of the 2017 World Workshop on the Classification of Periodontal and Peri-Implant Diseases and Conditions. J Periodontol. 2018 Jun; 89 Suppl 1:S237-S248.
9. **Borba VV, Zandman-Goddard G, Shoenfeld Y.** Prolactin and Autoimmunity. Front Immunol. 2018 Feb 12; 9:73.
10. **Sangeeta Devi Y, Halperin J.** Reproductive actions of prolactin mediated through short and long receptor isoforms. Mol Cell Endocrinol. 2014 Jan 25; 382(1):400-410.
11. **Krasselt M, Baerwald C.** Sex, Symptom Severity, and Quality of Life in Rheumatology. Clin Rev Allergy Immunol. 2019 Jun; 56(3):346-361.
12. **Tang MW, Garcia S, Gerlag DM, Tak PP, Reedquist KA.** Insight into the Endocrine System and the Immune System: A Review of the Inflammatory Role of Prolactin in Rheumatoid Arthritis and Psoriatic Arthritis. Front Immunol. 2017 Jun 23; 8:720.
13. **Fatima T, Khurshid Z, Rehman A, Imran E, Srivastava KC, Shrivastava D.** Gingival Crevicular Fluid (GCF): A Diagnostic Tool for the Detection of Periodontal Health and Diseases. Molecules. 2021 Feb 24; 26(5):1208.
14. **Tran TT, Ngo QT, Tran DH, Nguyen TT.** Effect of Two Nonsurgical Periodontal Treatment Modalities in Type 2 Diabetes Mellitus Patients with Chronic Periodontitis: A Randomized Clinical Trial. J Contemp Dent Pract. 2021 Nov 1; 22(11):1275-1280.

15. **Pal D, Nasim F, Chakrabarty H, Chakraborty A.** Non-surgical periodontal therapy: an evidence-based perspective. J Dent Panacea. 2021 ;3(2):1–4
16. **Baeza M., Morales A., Cisterna C., Cavalla F., Jara G., Isamitt Y., et al.** Effect of periodontal treatment in patients with periodontitis and diabetes: systematic review and meta-analysis. J. Appl. Oral Sci. 2020; 28.
17. **El-Wakeel NM, Shalaby ZF, Abdulmaguid RF, Elhamed SSA, Shaker O.** Local gingival crevicular fluid, synovial fluid, and circulating levels of prolactin hormone in patients with moderately active rheumatoid arthritis and stage III and IV periodontitis before and after non-surgical periodontal treatment-a controlled trial. Clin Oral Investig. 2023 Jun; 27(6):2813-2821.
18. **Rasheed HA, Al-Kuraishy HM, Al-Gareeb AI, Hussien NR, Al-Nami MS.** Effects of diabetic pharmacotherapy on prolactin hormone in patients with type 2 diabetes mellitus: Bane or Boon. J Adv Pharm Technol Res. 2019 Oct-Dec; 10(4):163-168.
19. **Silness J and Loe H.** Periodontal disease in pregnancy II. Correlation between oral hygiene and periodontal condition. Acta Odontol Scand. 1964; 22:112– 120.
20. **Loe H and Silness J.** Periodontal disease in pregnancy I. Prevalence and severity. Acta Odontol Scand.1963; 21:533–540.

# الملخص العربي


## الهدف من الدراسة:

إجراء الدراسة الحالية لتقييم الدور المحتمل لهرمون البرولاكتين في التسبب في التهاب اللثة من خلال تقييم مستوياته المحلية في اللثة لدى المرضى الذين يعانون من مرض السكري من النوع الثاني مع التهاب اللثة فى المرحلة الثالثة قبل وبعد العلاجات غير الجراحية مقارنة بالضوابط..

## خطة البحث

سوف يتم اجراء هذه الدراسة على مرضى مصابين بمرض السكرى والتهاب اللثة المستوى الثالث ، سيتم اختيارهم من العيادة الخارجية بكليه طب الأسنان للبنات جامعه الأزهر ، سوف يتم اختيار المرضى على اساس قواعد ومعايير محددة تتراوح من المحافظة على نظافة الفم وان يكونوا بصحة سليمة ولا بد من موافقة المريض على خطة العلاج .

## سوف يتم تقييم المرضى قبل اجراء الفحوصات:

- تقييم إكلينيكي.
- تاريخ طبي شامل.

## سوف يتم تقييم المرضى بعد ٣ شهور من المتابعه:

- فحص إكلينيكي.
- مستوى البرولاكتين
- قياس عمق الجيب اللثوي.

## المرحلة العلاجية:

**المجموعة الأولى:** أصحاء (التحكم).

**المجموعة الثانية:** مرضى المرحلة الثالثة من التهاب اللثة بدون مرض السكري

**المجموعة الثالثة:** المرحلة الثالثة من التهاب اللثة مع مرض السكري من النوع الثاني المنضبط

**المجموعة الرابعة:** المرحلة الثالثة من التهاب اللثة مع مرض السكري من النوع الثاني غير المنضبط

## المشرفون

**أ.د/ نجلاء محمد الوكيل**

أستاذ و رئيس قسم طب الفم وأمراض اللثة والتشخيص والأشعة
كلية طب الأسنان للبنات
جامعة الأزهر

**أ.د/ ألفت جميل شاكر**

أستاذ الكيمياء الحيوية الطبية والبيولوجيا الجزيئية

كلية الطب

جامعة القاهرة

**د/ زهراء محمد أحمد سعيد**

مدرس طب الفم وأمراض اللثة والتشخيص والأشعة
كلية طب الأسنان للبنات
جامعة الأزهر

# مستويات السائل اللثوي من هرمون البرولاكتين لدى مرضى السُكرى من النوع الثانى المصابين بالتهاب اللثة فى المرحلة الثالثة قبل وبعد العلاجات غير الجراحية

**خطه بحث**

**مقدمه إلى كلية طب الأسنان للبنات**

**جامعة الأزهر**

كجزء من مقومات الحصول علي درجه الماجستير

**في**

**طب الفم وأمراض اللثة والتشخيص والأشعة**

**مقدمه من**


**الطبيبة/ ساره إبراهيم حسين عيد**

بكالوريوس طب الفم والأسنان (٢٠١٧) جامعة الأزهر



كلية طب الاسنان للبنات

جامعة الأزهر

١٤٤٥هـ -٢٠٢٤م
