# Supplementary material for: Gingival crevicular fluid levels of prolactin in periodontitis patients with controlled versus uncontrolled type 2 diabetes. a controlled clinical trial
Source: Sci Rep. 2026 Sep 7;16:27945. doi: 10.1038/s41598-026-68268-1 (PMC13550468; doi:10.1038/s41598-026-68268-1)
Supplement: Supplementary file 3 [file 41598_2026_68268_MOESM3_ESM.docx]

**Title Page**

**Thematic Area:** Periodontics

**Title**: Effects of Non-Surgical Periodontal Therapy on Gingival Crevicular Fluid Prolactin Hormone Levels in Stage III Periodontitis Patients with Controlled and Uncontrolled Type 2 Diabetes

**Authors:**

**Sara Ibrahim Hussein Eid**	[sarah_ebrahem214474@azhar.edu.eg](mailto:sarah_ebrahem214474@azhar.edu.eg)	ORCID iD: 0009-0005-9936-6647

**Zahraa Mohamed Ahmed Saed**	[**zahraasaid.26@azhar.edu.eg**](mailto:zahraasaid.26@azhar.edu.eg)	ORCID iD: 0009-0005-7302-9773

**Olfat Gamel Shaker** [**olfatshaker@yahoo.com**](mailto:olfatshaker@yahoo.com) ORCID iD: 0000 0002 3031 3599

**Naglaa Mohamed El-Wakeel**	[**naglaelwakeel@gmail.com**](mailto:naglaelwakeel@gmail.com)	[**https://orcid.org/0000-0001-8169-841X**](https://orcid.org/0000-0001-8169-841X)

**Author Affiliations**

**Sara I. Hussein ^1^, Zahraa M. Ahmed^2^, Olfat G. Shaker ^3^, Naglaa El-Wakeel ^2*^**

1. General Dentist, Ministry of Health, Cairo, Egypt; Master student, Department of Oral Medicine, Periodontology, Oral Diagnosis and Radiology, Faculty of Dental Medicine for Girls, Al-Azhar University, Cairo, Egypt (Master’s Student)

Egyptian Ministry of Health, Cairo, Egypt.

2. Oral Medicine, Periodontology, Oral Diagnosis and Radiology Department, Faculty of Dental Medicine for Girls, Al-Azhar University, Cairo, Egypt
3. Medical Biochemistry and Molecular Biology, Faculty of Medicine, Cairo University, Cairo, Egypt.

***Corresponding author:**

**Sara Ibrahim Eid**

***General Dentist, Ministry of Health, Cairo, Egypt; Master student, Department of Oral Medicine, Periodontology, Oral Diagnosis and Radiology, Faculty of Dental Medicine for Girls, Al-Azhar University, Cairo, Egypt (Master’s Student)***

Egyptian Ministry of Health, Cairo, Egypt

**Email** sarah_ebrahem214474@azhar.edu.eg
